# Preparation and Evaluation of Zn_2_(BDC)_2_(DABCO)-β-CD MOF as a Dual-Drug Delivery Nanocomposite Containing Tetracycline and Indomethacin for Skin Wound Healing in BALB/c Mice

**DOI:** 10.5812/ijpr-167924

**Published:** 2026-06-14

**Authors:** Farya Mirzaei, Negar Motakef Kazemi, Naser Mohamadpour Dounighi, Mostafa Saffari, Sepideh Arbabi Bidgoli

**Affiliations:** 1 Department of Medical Nanotechnology, TeMS.C., Islamic Azad University, Tehran, Iran; 2 Department of Human Vaccines and Serum, Razi Vaccine and Sera Research Institute, Karaj, Iran; 3 Department of Pharmaceutics and Medical Nanotechnology, Branch of Pharmaceutical Sciences, Islamic Azad University, Tehran, Iran; 4 Department of Medical Toxicology and Pharmacology, TeMS.C., Islamic Azad University, Tehran, Iran

**Keywords:** Zn-β-CD MOF, Nanocomposite, Tetracycline, Indomethacin, Dual-drug Delivery

## Abstract

**Background:**

Skin wounds complicated by infection and inflammation represent a major clinical challenge and require effective delivery systems that combine antimicrobial and anti-inflammatory agents to promote healing. Nanobased systems, such as metal-organic frameworks (MOFs), are promising platforms for dual-drug delivery because of their high porosity, tunable structure, and biocompatibility.

**Objectives:**

This study aimed to synthesize a Zn_2_(BDC)_2_(DABCO)-β-cyclodextrin MOF nanocomposite using a hydrothermal method and to load indomethacin and tetracycline to develop a dual-drug delivery system that enhances the efficiency and therapeutic efficacy of topical treatment for skin wound healing.

**Methods:**

The morphology of the nanocomposite was characterized by scanning electron microscopy (SEM) and transmission electron microscopy (TEM). Zeta potential was measured using a Zetasizer; the crystalline structure was evaluated by X-ray diffraction (XRD); the physicochemical structure was assessed by Fourier transform infrared (FTIR) spectroscopy; and structural porosity before and after drug loading was analyzed by Brunauer-Emmett-Teller (BET) analysis. The system was then evaluated for in vitro cytotoxicity using the MTT assay, cytokine levels in blood samples from treated mice, and in vivo efficacy in mice, compared with the corresponding pure drugs.

**Results:**

The synthesized MOF exhibited high crystallinity. The average particle size was 277 nm for the blank nanocomposite and 545 nm for the drug-loaded nanocomposite, with a zeta potential of -2.6 mV. The drug-loading efficiency in phosphate-buffered saline (PBS; pH 7.4) was 28%, with biphasic release comprising an initial burst followed by sustained release over 144 hours. The BET surface area decreased after drug loading. In vitro studies showed that the drug-loaded nanocomposite was noncytotoxic to L929 fibroblast cells at all tested concentrations. The loaded MOF nanocomposite group showed significantly accelerated wound closure (97.5% on day 6) compared with all other groups (P < 0.05). Cytokine assays in mouse blood samples showed increased interleukin 10 (IL-10) and decreased interferon gamma (IFN-γ) in the group treated with the loaded nanocomposite compared with the control group.

**Conclusions:**

The Zn_2_(BDC)_2_(DABCO)-β-CD nanocomposite containing tetracycline and indomethacin exhibited noncytotoxic behavior, enhanced wound-healing efficacy, and sustained drug release under physiological conditions. These findings highlight its potential as a promising topical drug delivery system.

## 1. Background

Cutaneous wounds, particularly chronic and nonhealing wounds such as diabetic ulcers and infected skin injuries, pose major health care challenges that affect patients' quality of life and impose a substantial economic burden on health care systems (1). These wounds are complicated by infection and excessive inflammation, which delay healing. Therefore, effective wound management requires attention to multiple factors, including infection control, inflammation modulation, and tissue regeneration (2). Conventional wound treatments that rely on topical application face several challenges, including low penetration, burst release, inability to deliver multiple drugs simultaneously at a single site, rapid drug clearance, poor local retention, limited efficacy, and systemic adverse effects (3). Topical therapies that integrate antimicrobial and anti-inflammatory properties are essential for promoting healing (4). Conventional wound treatment approaches often rely on the topical application of single therapeutic agents, such as antibiotics for infection prevention or anti-inflammatory drugs for pain and inflammation management (5).

Tetracycline, a broad-spectrum antibiotic, has excellent antimicrobial activity against both gram-positive and gram-negative bacteria commonly associated with wound infections (6). In addition, tetracycline has matrix metalloproteinase (MMP)-inhibitory properties, which can reduce excessive extracellular matrix degradation in chronic wounds (7). Indomethacin, a nonsteroidal anti-inflammatory drug (NSAID), effectively reduces inflammation and pain through cyclooxygenase (COX) inhibition (8). Although inflammation is essential for the initiation of wound healing, excessive or prolonged inflammatory responses can impair tissue regeneration. Strategic modulation of inflammation using NSAIDs can optimize healing outcomes (9).

Nanocarriers enable localized sustained release and improve drug stability and efficacy (10). Metal-organic frameworks (MOFs) have been extensively investigated as drug delivery systems because of their tunable porosity and high loading capacity. For example, zinc-based MOFs such as ZIF-8 have been used to load antibiotics, including tetracycline, for antibacterial therapy (11).

MOFs, particularly zinc-based varieties functionalized with β-cyclodextrin (β-CD), are porous crystalline materials that have emerged as versatile drug delivery platforms. Their unique properties, including high surface area, tunable porosity, biocompatibility, improved hydrophilicity, host-guest drug inclusion, biological tissue interaction, and multidrug loading capacity, make them favorable carriers for controlled and targeted delivery (12, 13).

MOFs such as ZIF-8 are attractive because of their simple synthesis, small particle size, and well-established drug delivery applications. However, several considerations justified the selection of Zn_2_(BDC)_2_(DABCO)-β-CD in this study.

1. Drug loading versatility: ZIF-8 has hydrophobic internal surfaces and lacks intrinsic host-guest inclusion capability for hydrophobic molecules, unlike Zn-β-CD, which provides a dual-compartment loading mechanism.

2. Controlled dual release: ZIF-8 exhibits pH-sensitive degradation, which is beneficial for tumor therapy. In contrast, the Zn_2_(BDC)_2_(DABCO)-β-CD system provides diffusion-controlled release from MOF pores and potentially smoother release kinetics, which are advantageous for sustained topical therapy (14).

3. Structural stability and tunability: The Zn_2_(BDC)_2_(DABCO) framework offers larger pore dimensions than many ZIF systems, enables π-π interactions, provides better adaptability for postsynthetic modification such as β-CD integration, and supports the rational design of multidrug systems (15).

The choice of Zn_2_(BDC)_2_(DABCO)-β-CD was guided by functional design requirements rather than by simplicity of synthesis. Zinc-based frameworks structurally related to Zn_2_(BDC)_2_(DABCO) have been evaluated for single-drug delivery applications, mainly focusing on either antimicrobial or anticancer agents (13). However, these studies primarily investigated single-drug systems, and dual therapeutic strategies were not explored. Co-loading antibacterial tetracycline and anti-inflammatory indomethacin may synergistically enhance wound repair by combating infection and reducing local inflammation (16).

The novelty of this project is the simultaneous co-loading of tetracycline and indomethacin within a Zn_2_(BDC)_2_(DABCO)-MOF platform modified with β-cyclodextrin. No previous study has described β-CD modification of Zn_2_(BDC)_2_(DABCO) specifically to create dual loading compartments: one for hydrophilic antibiotic incorporation through coordination and hydrogen bonding and another hydrophobic cavity for indomethacin inclusion. This specific hybrid system has not previously been presented as a topical ointment formulation for wound healing with in vivo evaluation. Therefore, the novelty of the present study lies in the rational integration of 1) a zinc-based porous MOF matrix for tetracycline loading, 2) β-cyclodextrin functionalization for hydrophobic indomethacin encapsulation, and 3) a dual-action sustained-release topical platform targeting both bacterial infection and inflammation in wound healing.

Unlike previous studies that focused on either antibacterial or anti-inflammatory monotherapy, the present work introduces a multifunctional β-CD-modified Zn_2_(BDC)_2_(DABCO) nanocomposite engineered for synchronized dual-drug delivery, addressing 2 major pathological components of wound repair within a single nanocarrier system.

Zn_2_(BDC)_2_(DABCO) MOF (BDC = 1,4-benzenedicarboxylate; DABCO = 1,4-diazabicyclo[2.2.2]octane) has a pillared-layer structure with accessible pores suitable for drug encapsulation (17). The components of the MOF matrix create a heterogeneous microenvironment that confers distinct physicochemical characteristics influencing drug loading and release behavior.

Zinc is an essential trace element with a critical biological role in tissue repair. Therefore, partial degradation of the MOF and controlled Zn^2+^ release may synergistically enhance wound healing alongside tetracycline and indomethacin. The presence of zinc metal nodes and carboxylate linkages also generates polar coordination sites capable of hydrogen bonding and electrostatic interactions (11).

Regarding the Zn_2_(BDC)_2_(DABCO) framework, its internal pore environment is not purely hydrophobic. The terephthalate (BDC) aromatic rings introduce partially hydrophobic domains within the framework, which can promote π-π and hydrophobic interactions with drug molecules. The organic linker BDC and the DABCO ligand do not have inherent wound-healing activity; however, they contribute to the structural rigidity, porosity, and controlled degradation behavior of the framework (18).

The presence of zinc metal nodes and carboxylate linkages generates polar coordination sites capable of hydrogen bonding and electrostatic interactions. The incorporation of β-CD enhances nanocomposite performance through multiple mechanisms (19). This framework incorporates β-CD, a biocompatible cyclic oligosaccharide with a hydrophobic cavity suitable for the inclusion of lipophilic drugs such as indomethacin. The external surface of β-CD is hydrophilic because of the presence of multiple hydroxyl groups. This amphiphilic nature enhances the aqueous dispersibility of the overall nanocomposite while enabling selective host-guest complexation, controlling release kinetics, and improving bioavailability for combined antibacterial and anti-inflammatory therapy (20).

Tetracycline, which contains multiple hydroxyl and amide functional groups, is primarily associated with the MOF framework through hydrogen bonding and possible coordination interactions with Zn^2+^ centers (6). In contrast, indomethacin is preferentially encapsulated within the hydrophobic cavity of β-CD through host-guest inclusion (21).

This mixed chemical environment enables the system to accommodate both hydrophilic and hydrophobic drugs simultaneously while enhancing the antibacterial properties of the zinc-based nanocomposite and leveraging the synergistic effects of indomethacin and tetracycline in wound repair and healing.

## 2. Objectives

In this study, Zn_2_(BDC)_2_(DABCO)-β-CD MOF nanocomposites co-loaded with tetracycline and indomethacin were synthesized, formulated as a topical ointment, and evaluated in BALB/c mice in comparison with the pure drugs. Evaluation metrics included physicochemical properties, in vitro cytotoxicity assessed by the MTT assay, in vivo wound healing, and cytokine levels measured by enzyme-linked immunosorbent assay (ELISA).

## 3. Methods

### 3.1. Materials

Zinc acetate dihydrate (Zn(NO_3_)_2_2H_2_O, 99%), 1,4-benzenedicarboxylic acid (BDC, 98%), 1,4-diazabicyclo[2.2.2]octane (DABCO, 99%), β-cyclodextrin (β-CD, ≥ 97%), and ethanol were purchased from Merck Company (Germany). Tetracycline hydrochloride and indomethacin were obtained from Hakim Pharmaceutical Company, and distilled water was obtained from a reputable chemical supplier. All chemicals were used without further purification.

### 3.2. Synthesis of Zn_2_(BDC)_2_(DABCO)-Β-CD MOF

The MOF was synthesized using a modified hydrothermal method (22, 23). The molar ratio of Zn, BDC, DABCO, and β-CD was 2:2:1:1. Briefly, Zn(NO_3_)_2_2H_2_O (0.220 g, 2 mmol) and DABCO (0.035 g, 1 mmol) were dissolved in 20 mL of distilled water. BDC (0.167 g, 2 mmol) was dissolved in 5 mL of ethanol and added to the synthesis medium in a sterile 100-mL glass flask. Then, 0.1 g (1 mmol) of β-cyclodextrin was added to the mixture, after which the mixture became white and turbid. The mixture was stirred at 250 rpm and 90°C for 24 hours and then cooled to room temperature. The resulting white crystalline product was collected by centrifugation (20,000 rpm, 20 min) and washed 3 times with distilled water. The resulting paste was dried by lyophilization.

### 3.3. Drug Loading into the Zn_2_(BDC)_2_(DABCO)-Β-CD MOF Nanocomposite

Tetracycline and indomethacin were loaded into the Zn_2_(BDC)_2_(DABCO)-β-CD MOF using an immersion method (15). Ten milligrams of the synthesized MOF powder was added to 10 mL of distilled water and briefly stirred. Then, 5 mg of each drug (tetracycline and indomethacin) was added to the mixture, and the 100-mL glass flask was vacuumed and stirred at 250 rpm at room temperature for 24 hours in the dark. The drug-loaded MOF was collected by centrifugation (20,000 rpm, 20 min, 4°C) 3 times and dried by lyophilization. The drug-loaded MOF nanocomposite was incorporated into a petroleum jelly-based ointment at appropriate concentrations (24).

### 3.4. Characterization

#### 3.4.1. X-Ray Diffraction

X-ray diffraction (XRD) spectroscopy is a highly accurate, nondestructive method used to identify and investigate crystal structure and to determine phases and crystal lattice properties. In this study, the crystal phase and changes due to drug loading were recorded and investigated using a Philips PW1730 XRD instrument with Cu Kα radiation (λ = 1.5406 Å) over the 2θ range of 10 - 80°.

#### 3.4.2. Fourier Transform Infrared Spectroscopy

Fourier transform infrared (FTIR) analysis was used to identify substances, investigate chemical bonds and material structure, and confirm drug loading in the samples, including the nanocomposite containing both drugs, the nanocomposite before drug loading, tetracycline, and indomethacin. FTIR spectra were obtained using a Thermo Nicolet Avatar 380 FTIR spectrometer in the range of 4500 - 400 cm^-1^ using KBr pellets.

#### 3.4.3. Scanning Electron Microscopy and Transmission Electron Microscopy

Electron microscopy is a powerful tool for material analysis that uses an electron beam to achieve very high magnification and atomic-scale resolution. This method provides detailed information on internal structure, surface morphology, and particle size, particularly in nanocomposites. Size and morphological analyses were performed using scanning electron microscopy (SEM; SEM VEGA3, Czech Republic) and transmission electron microscopy (TEM; Philips EM208S, 100 kV, Netherlands).

#### 3.4.4. Zeta Potential

Zeta potential is a measure of the electrostatic stability of particles in solution. A near-zero or slightly negative zeta potential is usually beneficial for reducing topical toxicity and preventing unwanted adhesion to healthy cells. *Surface charge* was measured using a Malvern Zetasizer Nano ZS instrument.

#### 3.4.5. Brunauer-Emmett-Teller Analysis

Brunauer-Emmett-Teller (BET) analysis is an instrumental method for measuring surface porosity distribution, specific surface area, pore volume, and pore size, and for evaluating changes in surface area and adsorption capacity of nanocomposites after drug loading. Measurements were based on N_2_ adsorption-desorption isotherms at 77 K using a BET Mini II analyzer (Japan).

#### 3.4.6. In Vitro Drug Loading and Release Studies

Drug loading capacity (LC) and encapsulation efficiency (EE) were calculated using the following formulas:

LC (%) = (weight of loaded drug/weight of drug-loaded MOF) × 100

EE (%) = (weight of loaded drug/weight of initial drug) × 100

For this purpose, the weights of the dried nanocomposite, the dried drug-loaded nanocomposite, and the drug powders used in synthesis were measured using a digital balance and entered into the formulas (15).

To validate the loading data obtained from weight-based calculations, the residual amount in the supernatant after synthesis was also analyzed by ultraviolet-visible (UV-Vis) spectrophotometry and high-performance liquid chromatography (HPLC). After the loading process, the suspensions were centrifuged (10,000 rpm, 15 min, 4°C), and the concentration of unencapsulated free drug in the supernatant was quantified. UV-Vis measurements were performed, and HPLC analysis was used for improved accuracy and specificity. Established calibration curves showed good linearity within the tested concentration range (R^2^ > 0.99).

Release profiles were determined by measuring drug concentrations released from the nanocomposite into the supernatant using UV-Vis spectrophotometry (Shimadzu UV-1900i Plus, Japan) at λmax 265 nm for tetracycline (25) and 317 nm for indomethacin (21), using their calibration curves (R^2^ > 0.99). Chromatographic separation (Agilent 1200 Series, USA) was performed using a C18 reversed-phase column under isocratic conditions. The mobile phase consisted of acetonitrile and water adjusted to an appropriate pH and was delivered at a constant flow rate of 1.0 mL/min. Detection was performed at 265 nm for tetracycline and 317 nm for indomethacin. Calibration curves showed good linearity within the tested concentration range (R^2^ > 0.99).

In vitro drug release studies were performed using 10 mg of the drug-loaded nanocomposite in dialysis bags (molecular weight cutoff, 3.5 kDa) immersed in 100 mL of PBS (pH 7.4) at 37 ± 0.5°C under continuous magnetic stirring at 100 rpm (15). Assuming 30% loading, as reported in previous studies, 3 mg of the 10 mg drug-loaded nanocomposite powder would contain drug, corresponding to a drug concentration of 30 µg/mL. The value obtained in this study was 28%, which is close to or below the assumed value. This concentration is well below the reported aqueous solubility of tetracycline at physiological pH and remains below the saturation solubility of indomethacin at pH 7.4 because of its partial ionization. Thus, sink conditions were maintained throughout the release study.

At predetermined time intervals (0, 4, 8, 12, 24, 48, 72, 96, 120, and 144 hours), 5-mL aliquots were withdrawn and centrifuged (10,000 rpm, 15 min, 4°C), and 3 mL of the supernatant was analyzed. Then, 2 mL was mixed with 3 mL of fresh PBS and replaced to maintain a constant volume and continuous sink conditions. Released drug concentrations were quantified by UV-Vis spectrophotometry and confirmed by HPLC under the chromatographic conditions described above. All experiments were performed in triplicate, and results are expressed as mean ± standard deviation. Release kinetics were analyzed using zero-order, first-order, Higuchi, and Korsmeyer-Peppas mathematical models.

#### 3.4.7. In Vitro Cytotoxicity Assay

The MTT assay was performed using the L929 mouse fibroblast cell line. Cell viability was evaluated after exposure to 7 sample groups: 1) dual-drug-loaded nanocomposite, 2) blank nanocomposite, 3) tetracycline-loaded nanocomposite, 4) indomethacin-loaded nanocomposite, 5) tetracycline, 6) indomethacin, and 7) control (normal saline). Serial dilutions of 12.5, 25, 50, and 100 µg/mL were tested in triplicate for all concentrations (26). To minimize potential optical interference due to the intrinsic yellow color of tetracycline, drug-containing wells without cells were used as blanks, and background absorbance was subtracted from all measurements. Cells were washed before formazan solubilization to further eliminate residual drug-related absorbance.

#### 3.4.8. In Vivo Wound Healing Studies

##### 3.4.8.1. Animals and Ethical Approval

BALB/c mice (17 - 21 g) were obtained from the Laboratory Animal Center of Razi Vaccine and Serum Research Institute. Animals were housed under controlled environmental conditions (temperature, 22 ± 2°C; humidity, 55 ± 5%; 12-hour light/dark cycle) with free access to standard laboratory chow and water. All experimental procedures were approved by the Institutional Animal Ethics Committee (Approval No. IR.IAU.PS.REC.1404.045) and conducted in accordance with the Guide for the Care and Use of Laboratory Animals.

##### 3.4.8.2. Wound Creation

Mice were anesthetized by chloroform inhalation. The dorsal area was shaved and disinfected with 70% ethanol. Two to 3 full-thickness excisional wounds (1 cm) were created on the dorsal midline using a sterile surgical blade. Given that the commercial tetracycline ointment contains 3% active drug, the ointment was formulated using a 3:97 (w/w) ratio of nanocomposite to petroleum jelly. Subsequently, a sterile gauze dressing (1 × 1 cm) impregnated with the ointment was used to cover the wound area. The ointment was applied to the wound only once. After 6 days, blood samples were first collected from the retro-orbital venous plexus of the mice to minimize stress and prevent potential alterations in hematological parameters. Subsequently, the mice were individually weighed, and images of the dorsal wound area were captured for further analysis.

Animals were randomly divided into 5 groups (n = 3 per group): group 1, MOF-TC-Indo; group 2, blank MOF; group 3, commercial indomethacin; group 4, commercial tetracycline; and group 5, control, untreated wounds without drug. For safety assessment, body weight was measured at the end of the study.

Wound areas were photographed and measured on days 0 and 6 using ImageJ software. The wound closure percentage was calculated as follows (27):

$$\mathrm{Wound}\mathrm{closure}\left(\%\right)=\frac{A_{0}-A_{t}}{A_{0}}\times 100$$

where A_0_ is the initial wound area and A_t_ is the wound area at time t.

##### 3.4.8.3. Cytokine Analyses

An important function of NSAIDs in the wound-healing process is the activation of a cross-regulatory mechanism involving opposing cytokines, including the inhibition of signal transduction and Janus kinase-STAT signaling by suppressors of cytokine signaling. IFN-γ, a major activator of macrophages, inhibits 2 key anti-inflammatory functions of IL-10: suppression of cytokine production and major histocompatibility complex class II expression (28).

Accordingly, IFN-γ and IL-10 were evaluated in animal blood samples to compare the effectiveness of the synthesized constructs with pure drugs. On day 6, blood samples from the animals (n = 3 per group) were collected, and cytokines (IFN-γ and IL-10) were analyzed using ELISA kits according to the manufacturer's protocols.

## 4. Results

### 4.1. X-Ray Diffraction

**Figure 1.**
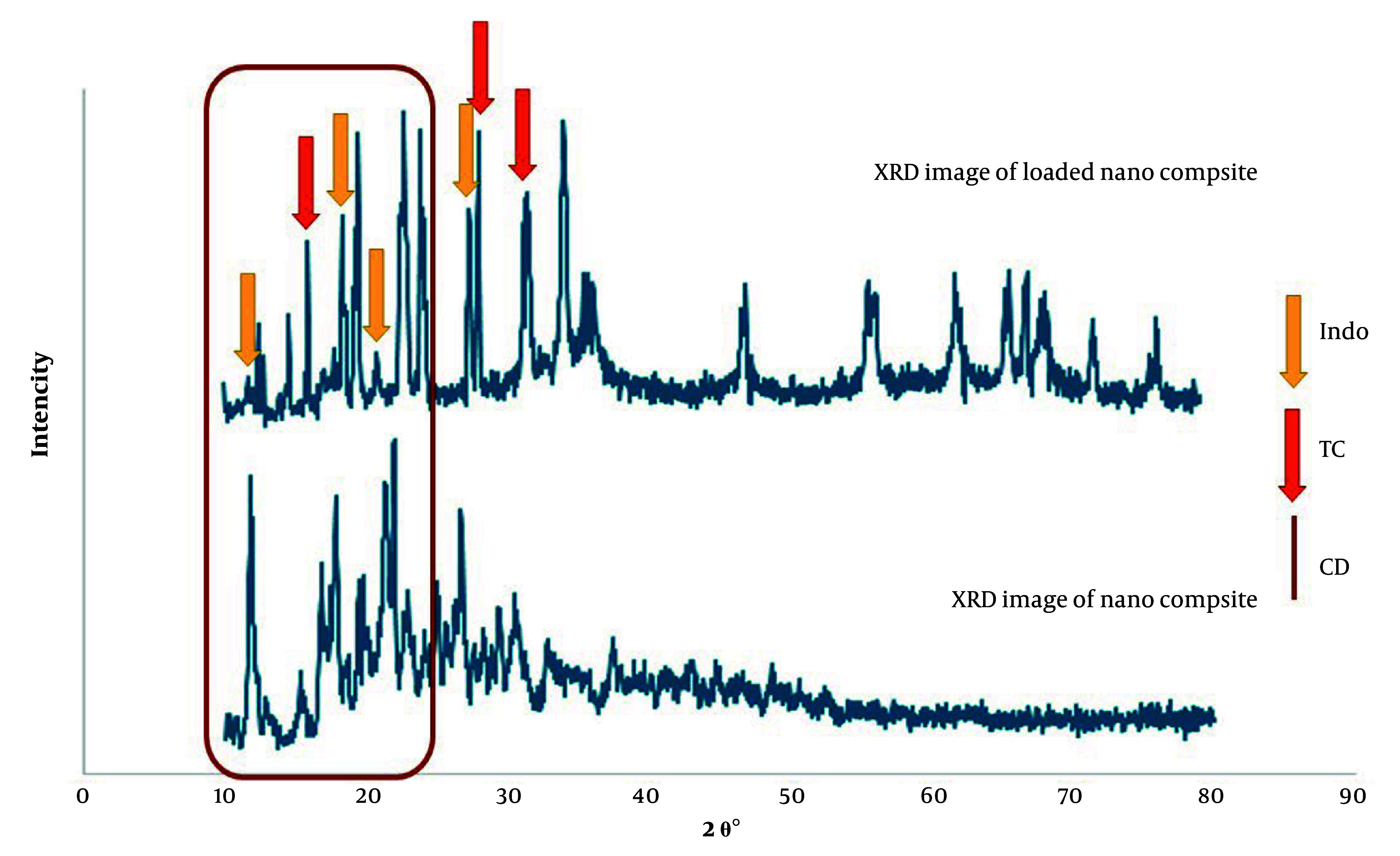
X-ray diffraction (XRD) graph of the Zn_2_(BDC)_2_(DABCO)-β-CD MOF nanocomposite before and after drug loading.

The XRD pattern of the synthesized Zn_2_(BDC)_2_(DABCO) MOF (Figure 1) showed characteristic diffraction peaks at 2θ = 11.78°, 12.94°, 15.26°, 16.82°, 17.82°, 21.9°, and 24.94°, confirming the pillared-layer structure and high crystallinity (Figure 1A) (15). The presence of characteristic peaks at 10.2°, 12.46°, 18.46°, and 22.9°, corresponding to β-cyclodextrin, indicated the presence of cyclodextrin in crystalline and semicrystalline forms within the stabilized MOF structure. Peaks at 10.2°, 15.98°, 22.9°, and 31.58° indicated the presence of tetracycline in the MOF matrix. Drug loading resulted in a slight reduction in peak intensity at 11.74°, 17.86°, 22.06°, and 27.1°, suggesting occupation of MOF pores by indomethacin.

The reduced intensity and slight increase in the width of some peaks (broadening) in the pattern of the loaded nanocomposite indicated a decreased degree of crystallinity (29) and minor structural disorder due to drug penetration into the pores and framework of the MOF (22). Preservation of the main peaks and the absence of new peaks indicated that the MOF crystal structure was not destroyed and that the drugs only slightly interfered with its network structure. These findings confirm successful loading of tetracycline and indomethacin into the Zn_2_(BDC)_2_(DABCO)-β-CD MOF nanocomposite while preserving the MOF structure.

### 4.2. Fourier Transform Infrared Spectroscopy

**Figure 2.**
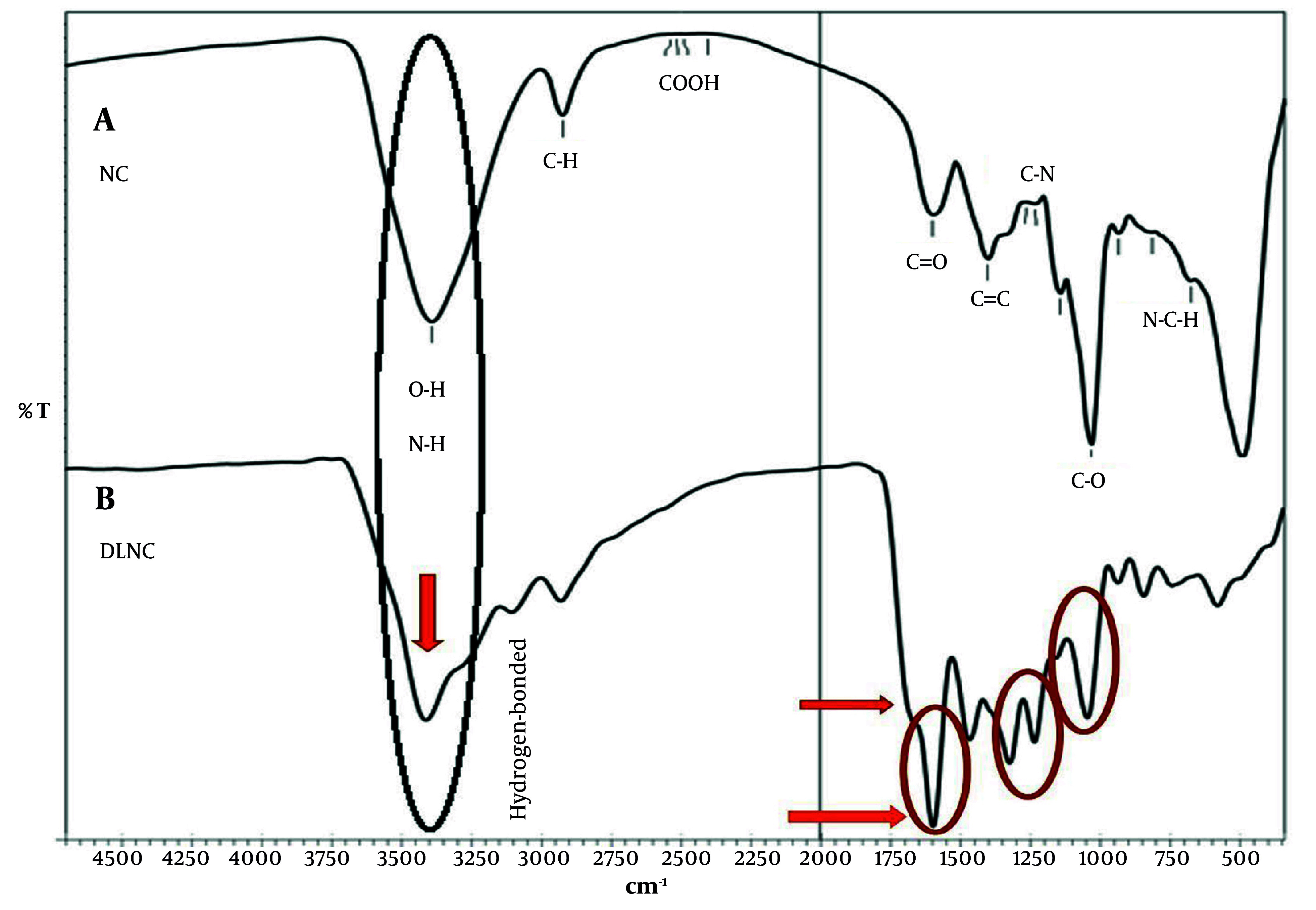
FTIR graph of the Zn_2_(BDC)_2_(DABCO)-β-CD MOF nanocomposite before (A) and after (B) loading tetracycline and indomethacin.

The FTIR spectra of the collected samples were recorded within 400 - 4500 cm^-1^. Figure 2 compares the FTIR spectra of the Zn_2_(BDC)_2_(DABCO)-β-CD MOF nanocomposite and the tetracycline-indomethacin-Zn_2_(BDC)_2_(DABCO)-β-CD MOF nanocomposite (Figure 2A and B, respectively).

The main peaks observed in the Zn_2_(BDC)_2_(DABCO)-β-CD MOF nanocomposite (Figure 2A) included a broad absorption band in the range of 3200 - 3600 cm^-1^, related to O-H stretching vibrations originating from β-CD and physically adsorbed moisture. Weak N-H stretching bands in this region corresponded to the presence of the DABCO ligand in the MOF structure. Peaks at 2920 - 2950 cm^-1^ were related to C-H stretching vibrations of the aliphatic groups of the BDC and DABCO ligands. Peaks at 1580 - 1650 cm^-1^ were related to C=O bond vibrations in carboxylate groups and C=C stretching vibrations of the aromatic rings of the BDC ligand, confirming coordination with Zn^2+^ ions in the MOF framework. Peaks at 1380 - 1430 cm^-1^ were related to C-N stretching vibrations associated with DABCO. Absorption bands in the 1000 - 1150 cm^-1^ region were attributed to C-O stretching vibrations, indicating the presence of the main groups constituting the MOF and cyclodextrin. Bands at 1582, 1136, 1100, 819, and 746 cm^-1^ were attributed to the N-C-H DABCO conformation in the pure MOF structure (30).

After loading tetracycline and indomethacin, noticeable changes were observed in spectrum B. The drug-loaded MOF exhibited characteristic shifts and reduced intensity in bands of both tetracycline (1720 cm^-1^, C=O stretching) and indomethacin (1700 cm^-1^, C=O stretching of carboxylic acid), confirming successful dual-drug encapsulation.

Some peaks in the 1300 - 1200 and 1100 - 1000 cm^-1^ regions, related to C-O and C-N vibrations, showed slight broadening and increased intensity. The O-H/N-H band in the 3200 - 3500 cm^-1^ region broadened because of strong hydrogen-bonding interactions between the hydroxyl groups of tetracycline and indomethacin and β-cyclodextrin, which are related to the stretching vibrations of OH and NH groups. Changes were also observed in the 1600 - 1750 cm^-1^ region, related to carbonyl and COOH groups. These observations suggest successful simultaneous drug loading, mainly through physical adsorption, hydrogen bonding, and host-guest interactions within the MOF pores, rather than structural degradation of the framework.

### 4.3. Electron Microscopy

**Figure 3.**
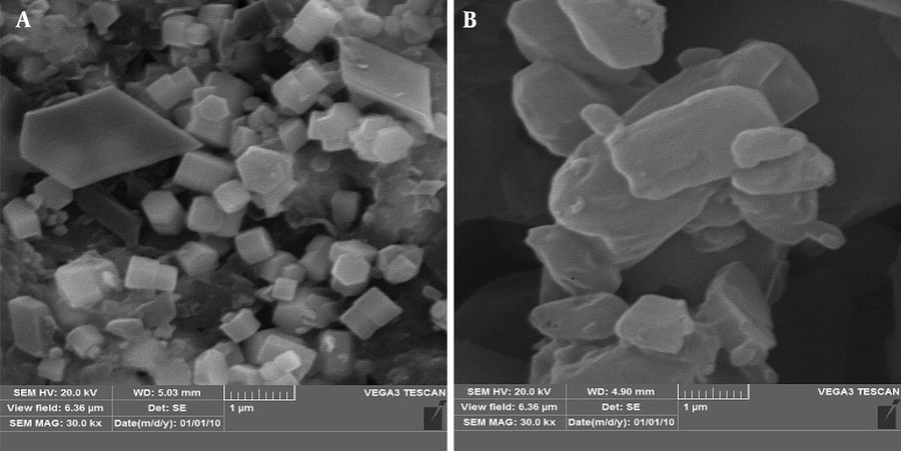
Scanning electron microscopy (SEM) images of the Zn_2_(BDC)_2_(DABCO)-β-CD MOF nanocomposite (A) and drug-loaded Zn_2_(BDC)_2_(DABCO)-β-CD MOF nanocomposites (B).

**Figure 4.**
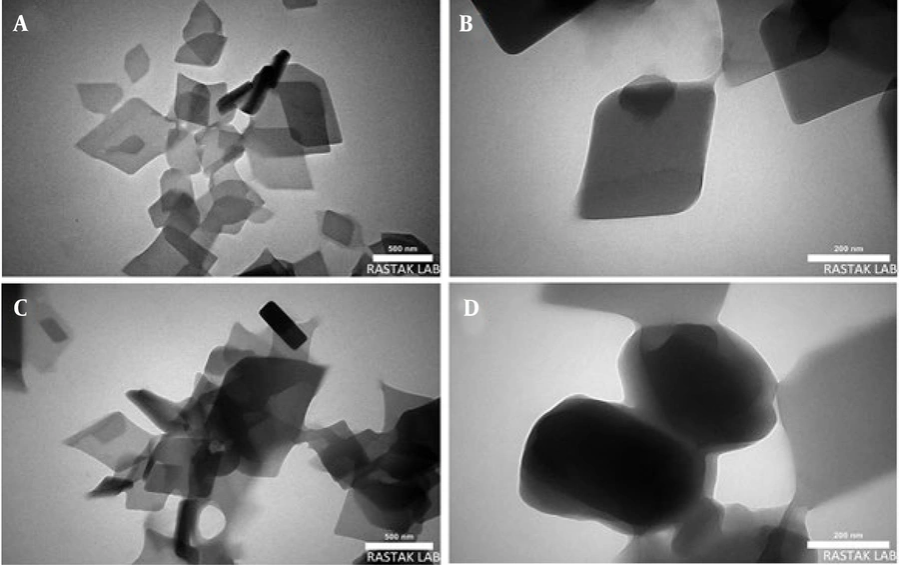
Transmission electron microscopy (TEM) images of the Zn_2_(BDC)_2_(DABCO)-β-CD MOF nanocomposite before drug loading (A) and after drug loading (B) at 200- and 500-nm scales.

**Figure 5.**
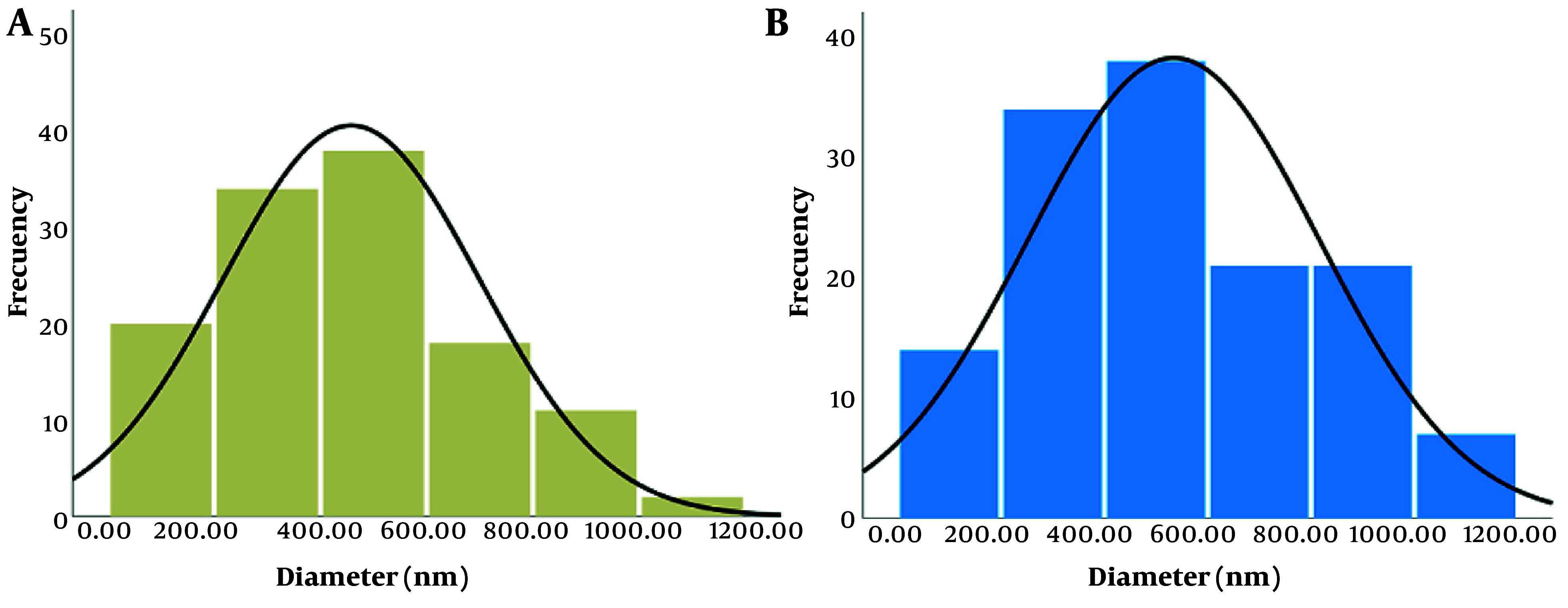
Histogram results from transmission electron microscopy (TEM) of the Zn_2_(BDC)_2_(DABCO)-β-CD MOF nanocomposite (A) and drug-loaded Zn_2_(BDC)_2_(DABCO)-β-CD MOF nanocomposites (B). Both graphs show a normal (Gaussian) distribution. The peak shifted from approximately 400 nm before loading to approximately 600 nm after loading, which may indicate an increase in particle size after loading.

SEM images showed that the synthesized MOF consisted of uniform trapezoidal cube-shaped crystals with an average particle size of ≤ 1 µm (Figure 3). After drug loading, particles appeared more aggregated, with slightly rougher surfaces. TEM analysis of ultrasonically dispersed samples showed individual nanoparticles with an average diameter of 200 - 700 nm (Figure 4), suitable for topical application and cellular uptake. Histogram graphs of the Zn_2_(BDC)_2_(DABCO)-β-CD MOF nanocomposites and drug-loaded Zn_2_(BDC)_2_(DABCO)-β-CD MOF nanocomposites are presented in Figure 5. The achieved nanoparticle size of approximately 500 nm is optimal for topical applications. Nanoparticles in this size range can penetrate the stratum corneum and reach deeper wound-bed tissues, enhancing drug bioavailability (31).

### 4.4. Zeta Potential

**Figure 6.**
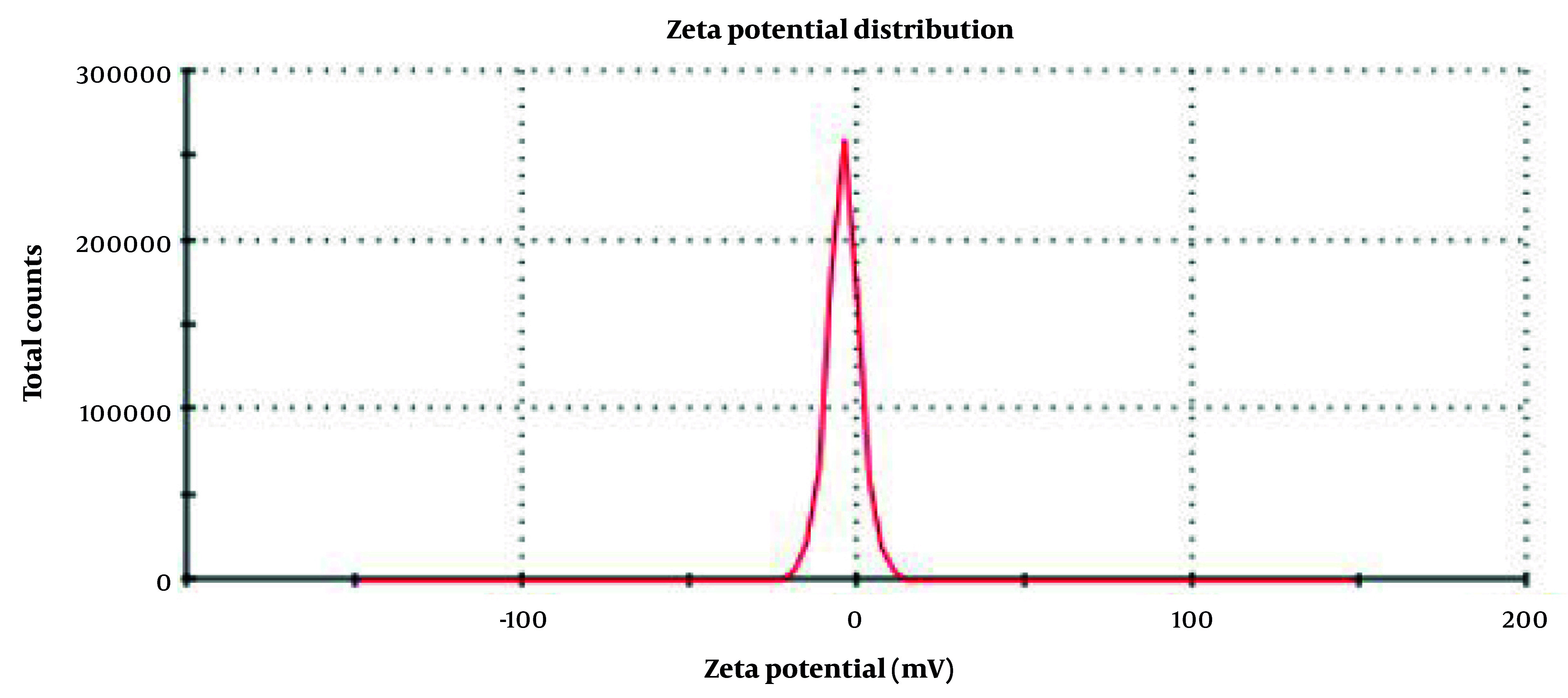
*Surface charge* (zeta potential) of the loaded Zn_2_(BDC)_2_(DABCO)-β-CD MOF nanocomposite. A slightly negative surface charge, which may be less irritating than a positive charge, also contributes to the stability of the nanocomposite in suspension.

Zeta potential measurements showed a negative surface charge (-2.6 mV) (Figure 6), which is beneficial for topical use and potential cellular interactions without skin irritation. The negative zeta potential provides electrostatic repulsion that prevents excessive aggregation while allowing interactions with positively charged wound components.

### 4.5. Brunauer-Emmett-Teller Analysis

**Figure 7.**
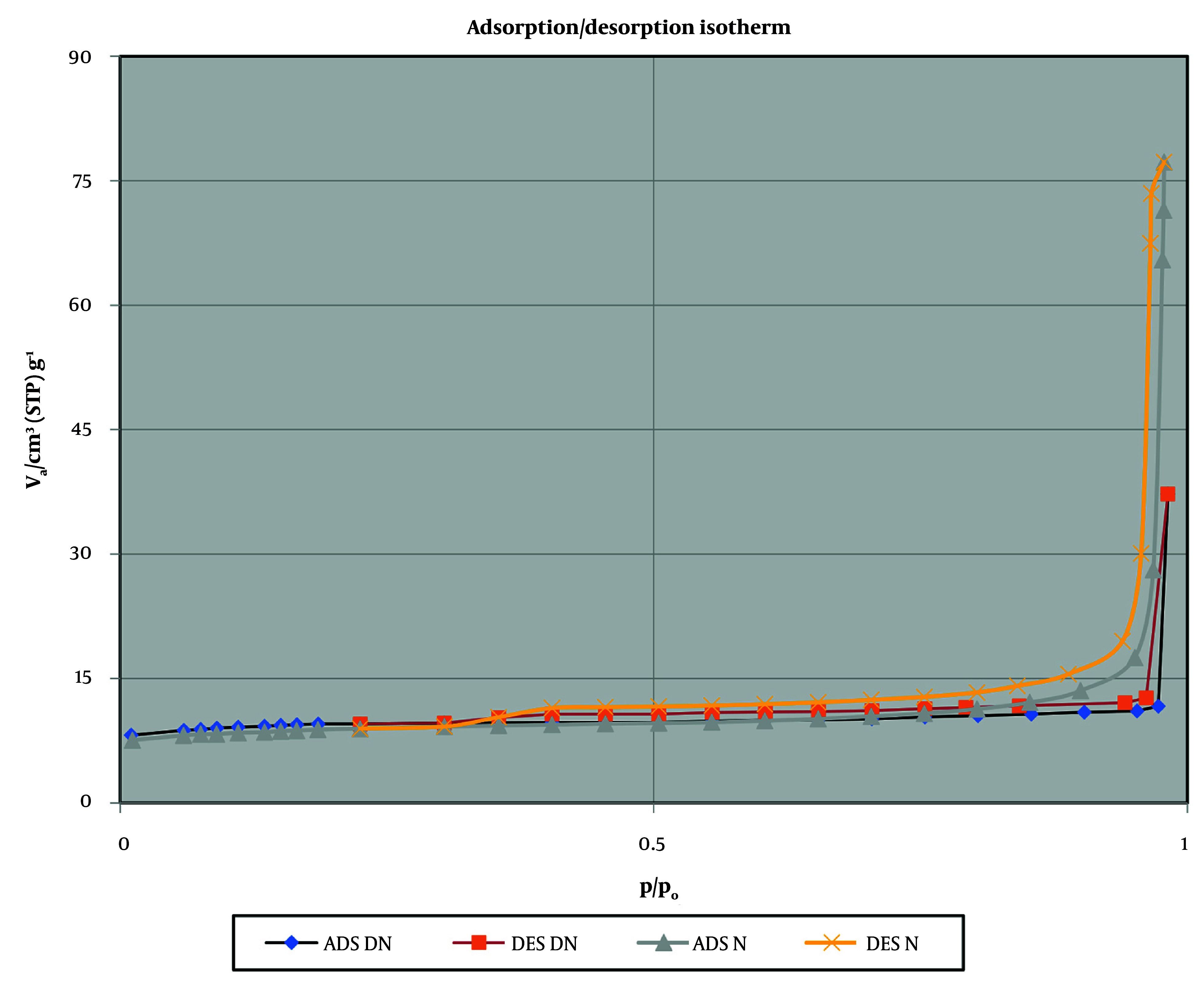
BET adsorption (ADS)/desorption (DES) isotherm of blank Zn_2_(BDC)_2_(DABCO)-β-CD MOF nanocomposite (N; blue and orange lines) and drug-loaded Zn_2_(BDC)_2_(DABCO)-β-CD MOF nanocomposites (DN; gray and yellow lines).

The nitrogen adsorption-desorption isotherm exhibited type II behavior, characteristic of microporous materials (18). According to IUPAC characterization, adsorption at 25°C, the presence of a type II adsorption isotherm (Figure 7), and a type H3 hysteresis loop indicate that the interior of β-CD may function as a gas host at the surface and in the channels. However, the Langmuir fit (R^2^ = 0.9994) indicates predominantly monolayer and superficial behavior. The BET surface area of the pristine MOF was 80.224 m^2^/g, with a pore volume of 0.120 cm^3^/g and an average pore diameter of 15.816 nm. After drug loading, the surface area decreased to 32.107 m^2^/g, confirming pore occupation by drug molecules. These changes confirm effective drug loading in the nanocomposite and pore filling, which affects drug release properties and structural stability.

### 4.6. Drug Loading and Encapsulation Efficiency

The measured weights were used in the formulas for encapsulated capacity (EC), encapsulation efficiency (EE), and drug loading capacity (LC), and the results are presented below (15).

Encapsulated capacity:

EC (%) = (drug in nanocomposite [mg] / drug-loaded nanocomposite [mg]) × 100

EC (%) = 3.89 mg / 13.89 mg × 100 = 28.0%

Encapsulation efficiency:

EE (%) = (drug in nanocomposite [mg] / drug initially used [mg]) × 100

EE (%) = 3.89 mg / 5 mg × 100 = 77.8%

Drug loading capacity:

LC (%) = (drug in nanocomposite [mg] / nanocomposite [mg]) × 100

LC (%) = 3.89 mg / 10 mg × 100 = 38.9%

According to these results, more than 77% of the drugs used were incorporated, and nearly half of the nanocomposite capacity was occupied by the drugs. Therefore, the nanocomposite structure showed high capability and appropriate capacity for successful and effective simultaneous loading of both drugs.

The differential loading of tetracycline and indomethacin reflects distinct drug-MOF interactions (32). The complementary loading mechanisms enabled efficient dual-drug encapsulation without competitive exclusion, representing a major advantage over conventional single-cavity carriers.

#### 4.6.1. In Vitro Drug Release Kinetics

**Figure 8.**
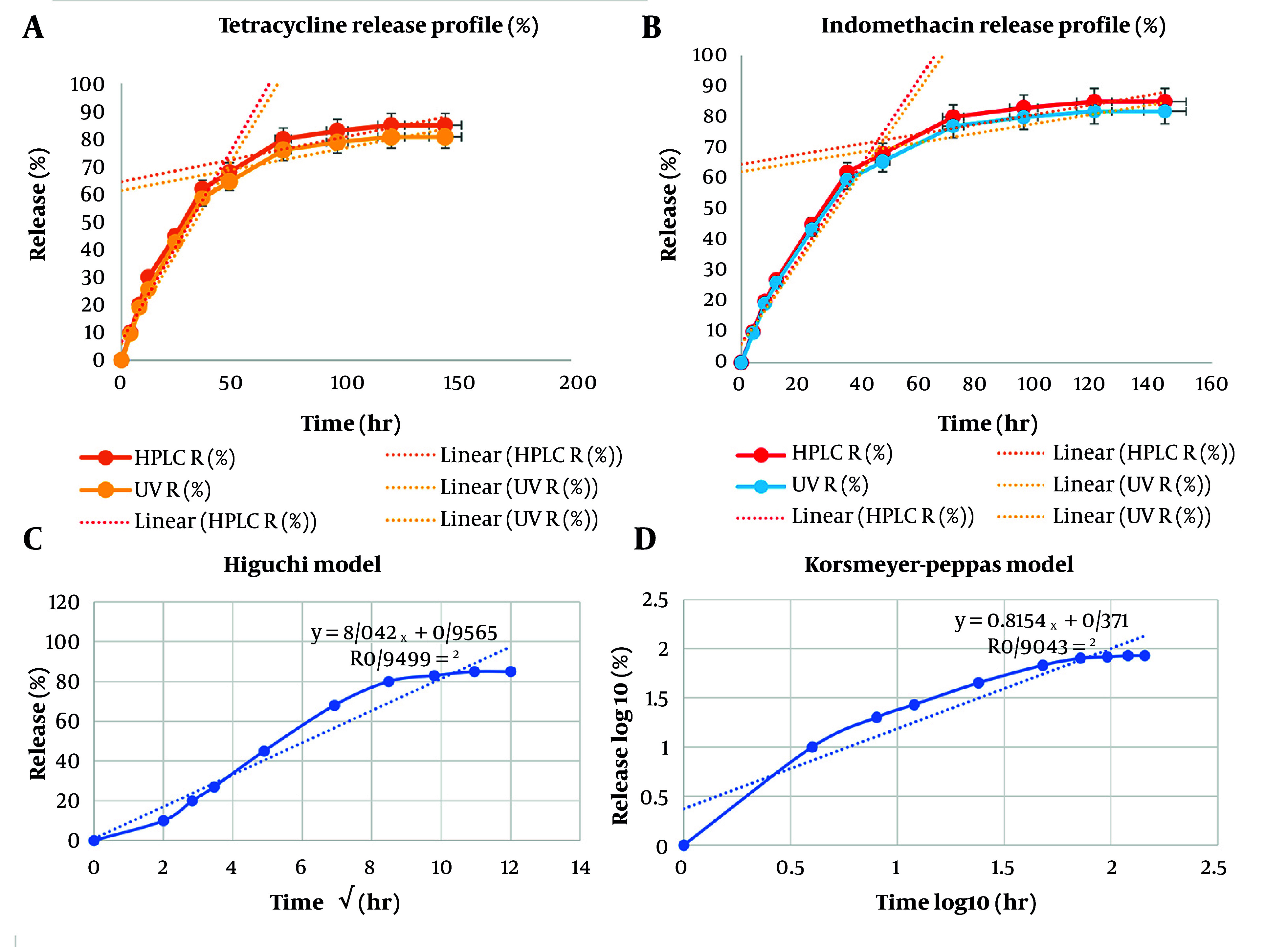
A, Schematic representation of tetracycline release from the nanocomposite by methods UV spectroscopy and HPLC, showing the slopes corresponding to the rapid and sustained release phases. B, Schema tic representation of Indomethacin release from the nanocomposite by methods UV spectroscopy and HPLC, showing the slopes corresponding to the rapid and sustained release phases. C, Diagrams related to the release profile analysis in Model Higushi. In this model, the slope of the equation will be equal to the value of "K". D, Diagrams related to the release profile analysis in Model Korsmeyer-Peppas. In this model, the slope of the equation will be equal to the value of "n" and y-intercep will be "K".

Drug release profiles in PBS (pH 7.4) showed sustained release characteristics over 144 hours (Figure 8A and B).

Both drugs exhibited biphasic release patterns, with an initial burst release within the first 36 hours followed by sustained release reaching more than 80% by 144 hours.

Release kinetics modeling results were used to identify the appropriate release equation for the nanocomposite. The R^2^ value, or linearity and goodness of fit of the data for each equation, was calculated. The highest R^2^ value was considered to represent the most appropriate release kinetics model for the system (15). The R^2^, K, and n values for different release models are presented in Table 1.

**Table 1.** Results From Calculating R^2^, K, and N to Determine the Drug Release Model from Nanocomposites

Items	Korsmeyer-Peppas model	Higuchi model	First-order kinetics	Zero-order kinetics
**R^2^**	0.9043	0.9499	0.4926	0.8205
**K**	0.371	8.042	0.0189	0.563
**n**	0.8154	-	-	-

The Higuchi model showed the best fit (R^2^ = 0.9499; Figure 8C), suggesting diffusion-controlled release. The Korsmeyer-Peppas model also showed an acceptable fit (R^2^ = 0.90, n = 0.81; Figure 8D), indicating non-Fickian or anomalous diffusion involving both diffusion and relaxation.

The sustained release profile is advantageous for wound-healing applications because it provides prolonged therapeutic effects while minimizing dosing frequency.

The biphasic release profile observed for both drugs is characteristic of MOF-based delivery systems (14). The initial burst release during the first 36 hours likely originated from surface-adsorbed drugs and drugs in easily accessible pores near the particle exterior. This rapid release provides immediate therapeutic concentrations for infection control and inflammation reduction. The gradual release pattern is consistent with gradual drug diffusion from the MOF pores and weak interactions between the drugs and the MOF structure, confirming the potential use of this nanocomposite as an efficient drug carrier for topical treatments (33).

### 4.7. In Vitro Cytotoxicity Assays

**Figure 9.**
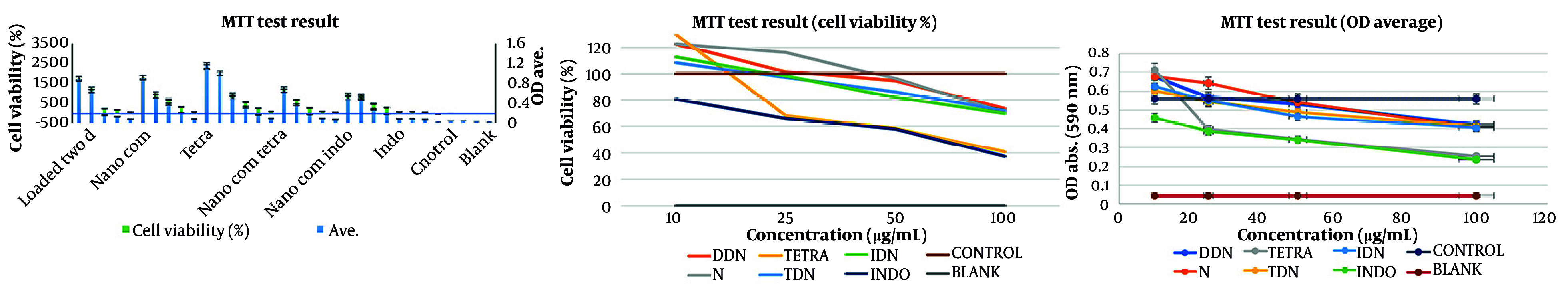
MTT assay results in L929 fibroblast cells for Zn_2_(BDC) _2_(DABCO)-β-CD MOF nanocomposite (N), drug-loaded Zn_2_(BDC) _2_(DABCO)-β-CD MOF nanocomposites as a dual-drug-loaded nanocomposite (DDN), tetracycline-loaded nanocomposite (TDN), indomethacin-loaded nanocomposite (IDN), and pure drugs tetracycline (TETRA) and indomethacin (INDO). Cells treated with water for injection (WFI) served as the untreated control, and blank wells contained no cell line. All samples were incubated for 24 hours at 37°C.

Mean results were obtained, and the percentage of cell viability in each sample group was calculated based on the tested concentrations. The corresponding graphs were generated (Figure 9).

For pure tetracycline and indomethacin, indomethacin at higher concentrations caused a greater reduction in viable cells. Although pure tetracycline showed higher absorbance than the nanocomposite-loaded samples, this observation cannot be conclusively attributed to biological activity without targeted control experiments.

The blank Zn_2_(BDC)_2_(DABCO)-β-CD MOF nanocomposite did not cause toxicity at any concentration, and the percentage of cell viability was not less than 70%, indicating high biocompatibility. All nanocomposites, both blank and loaded, showed similar results, indicating a lack of toxicity and supporting the reliability of the results obtained at the examined concentrations. In the report published by Mosmann in 1983, very high absorbance values in the MTT assay may indicate a positive effect of the sample on cell proliferation (26).

The drug-containing nanocomposite samples were also nontoxic and stimulated healthy cell proliferation, suggesting a protective effect and controlled drug release by the nanocomposite.

### 4.8. In Vivo Wound Healing Efficacy

#### 4.8.1. Macroscopic Wound Closure

**Figure 10.**
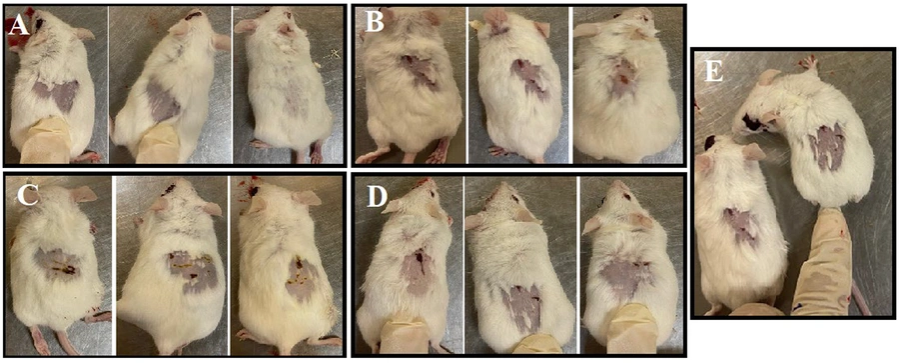
Photograph of the mice on the sixth day after intervention. (A) Group 1, Zn_2_(BDC) _2_(DABCO)-β-CD MOF nanocomposite containing both tetracycline and indomethacin; (B) group 2, Zn_2_(BDC) _2_(DABCO)-β-CD MOF nanocomposite before drug loading; (C) group 4, tetracycline; (D) group 3, indomethacin; and (E) group 5, control group with untreated wounds.

Visual assessment showed accelerated wound healing in the MOF-TC-Indo group compared with all other groups (Figure 10).

**Figure 11.**
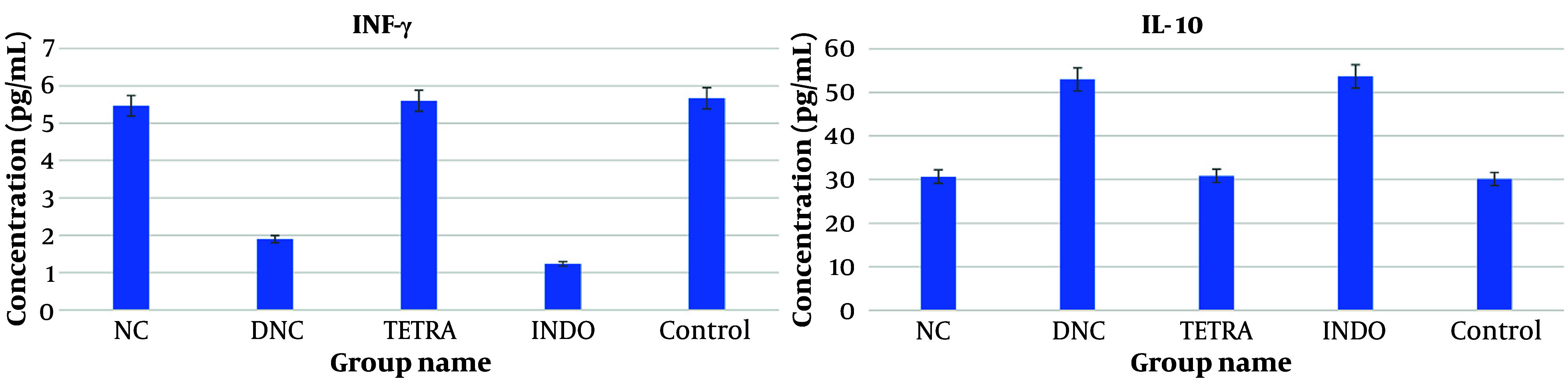
Results of measuring inflammatory and anti-inflammatory cytokine levels in blood samples from mice on day 6 after completion of the designed treatment period. Group NC, Zn_2_(BDC)_2_(DABCO)-β-CD MOF nanocomposite before drug loading; group DNC, Zn_2_(BDC)_2_(DABCO)-β-CD MOF nanocomposite containing both tetracycline and indomethacin; group TETRA, tetracycline; group INDO, indomethacin; and control, untreated wounds.

Quantitative analysis of wound closure percentages using ANOVA (Table 2) showed that the MOF-Tetra-Indo group achieved significantly superior wound closure compared with the control group (P = 0.006), the pure indomethacin group (P = 0.25), and the pure tetracycline group (P = 0.011). Notably, the nanocomposite outperformed even the pure drug groups, demonstrating the synergistic therapeutic benefits of MOF-mediated dual-drug delivery. These results require further investigation with a larger number of animals and more intervention groups.

**Table 2.** In Vivo Wound Healing Efficacy in Mice as the Animal Model

Group	Day 0 Wound Closure (%)	Day 0 Weight (g)	Day 6 Wound Closure (%)	Day 6 Weight (g)
**MOF-Tetra-Indo**	0.0	19.33	97.5	22
**Blank MOF**	0.0	19.66	72.4	22
**Indomethacin pure drug**	0.0	19.66	86.7	22
**Tetracycline pure drug**	0.0	19.66	72.7	21.6
**Control**	0.0	19.66	61.5	20

#### 4.8.2. Effects on Inflammatory and Anti-Inflammatory Cytokine Levels

ELISA analysis of ocular sinus vein blood samples on day 6 using ANOVA revealed the results shown in Table 3 and Figure 11.

**Table 3.** IFN-Γ and IL-10 Cytokine Test Results in Mouse Blood Samples After 6 Days

Cytokine	Sample	MOF-Tetra-Indo	Blank MOF	Indomethacin Pure Drug	Tetracycline Pure Drug	Control
**IFN-γ (pg/mg protein)**	Try 1	2	5	1	5.8	6
**IFN-γ (pg/mg protein)**	Try 2	1.9	5.6	1.2	6	6
**IFN-γ (pg/mg protein)**	Try 3	1.8	5.8	1.5	5	5
**IFN-γ (pg/mg protein)**	Average	1.9	5.466667	1.233333	5.6	5.666667
**IFN-γ (pg/mg protein)**	SD	0.1	0.42	0.25	0.52	0.58
**IFN-γ (pg/mg protein)**	Range	0.2	0.8	0.5	1.0	1.0
**IL-10 (pg/mg protein)**	Try 1	63	31	62	31	30
**IL-10 (pg/mg protein)**	Try 2	51	31	51	31.5	31
**IL-10 (pg/mg protein)**	Try 3	45	30	48	30	29.4
**IL-10 (pg/mg protein)**	Average	53	30.66667	53.66667	30.83333	30.13333
**IL-10 (pg/mg protein)**	SD	9.7	0.557	7.64	0.76	0.8
**IL-10 (pg/mg protein)**	Range	18	1.0	14.0	1.5	1.6

The MOF-Tetra-Indo group showed a significant change in pro-inflammatory cytokine levels, similar to the pure indomethacin group, and compared with all other groups (P < 0.05), indicating superior efficacy.

Measurement of inflammatory factors showed a significant decrease in IFN-γ and an increase in IL-10 in the nanocomposite containing both drugs, indicating the anti-inflammatory effects of the drug delivery system and effective relief of local inflammation.

The MOF-Tetra-Indo and commercial tetracycline groups showed significant antibacterial effects, with the nanocomposite demonstrating superior bacterial load reduction. This enhanced antimicrobial efficacy likely resulted from sustained tetracycline release and the intrinsic antimicrobial properties of the zinc-based MOF.

To assess safety, no significant body weight loss or major organ histological alterations were observed in treated animals.

## 5. Discussion

This study successfully developed a novel Zn_2_(BDC)_2_(DABCO)-β-CD MOF nanocomposite for the dual delivery of tetracycline and indomethacin with prolonged local release. The system demonstrated superior wound-healing efficacy compared with commercial drug formulations in a BALB/c mouse model. These findings highlight the potential of MOF-based drug delivery systems to provide controlled release and synergistic antibacterial and anti-inflammatory effects in complex wound management. Incorporation of β-CD into the Zn_2_(BDC)_2_(DABCO) framework conferred several advantageous properties that improved nanocomposite performance, including 1) biocompatibility with intrinsic antimicrobial activity (11), 2) a pillared-layer architecture that enhanced drug loading, 3) chemical stability, and 4) biodegradability. The hydrophobic cavity of β-CD may improve solubility and stability (34, 35), increase surface biocompatibility, and provide additional drug-binding sites, thereby contributing to a higher loading capacity.

A critical concern in the clinical use of tetracycline is the development of microbial resistance (7). Uncontrolled or subtherapeutic antibiotic exposure can accelerate resistance development (6). This study presents the successful formulation of a Zn_2_(BDC)_2_(DABCO)-β-CD MOF nanocomposite co-loaded with tetracycline and indomethacin and incorporated into a topical ointment for dual antibacterial and anti-inflammatory wound therapy. Importantly, this dual-delivery approach offers a promising combination-therapy strategy to enhance wound healing while potentially reducing the risk of antimicrobial resistance through controlled localized therapy, reduced dosing frequency, and a potential synergistic metal-ion effect (21).

Physicochemical characterization confirmed the preservation of structural integrity after dual-drug loading, and biological evaluation demonstrated enhanced wound-healing efficacy with minimal cytotoxicity. However, several mechanistic and comparative aspects warrant further discussion.

In this project, cubic drug-loaded nanocomposites with a particle size of approximately 500 nm and a zeta potential of -2.6 mV demonstrated good penetration potential and low toxicity, supporting their suitability for topical drug delivery. However, some CD-MOF systems reported in the literature exhibit rhombic dodecahedral or irregular polyhedral morphologies, typically 100 - 300 nm, depending on the synthesis solvent and metal source. Particles in the 400 - 600 nm range are often advantageous for localized topical therapy because they limit systemic diffusion while enabling sustained drug deposition at the wound site (36).

The measured negative surface charge is close to neutrality and is insufficient to provide strong electrostatic repulsion between particles; however, it likely supports tetracycline loading through hydrodynamic bonding and coordination interactions with Zn^2+^ nodes (13). The slightly negative zeta potential (-2.6 mV) suggests that steric stabilization rather than electrostatic stabilization may predominate in this system. Previously reported cyclodextrin-based MOFs, such as γ-CD-MOFs, or zinc-based MOFs, such as ZIF-8, typically exhibit higher absolute zeta potentials (-10 to -30 mV) (11, 33). Compared with these systems, our formulation has a lower electrostatic stabilization capacity. To improve stability in future work and in other formulations, such as colloidal oral or injectable formulations, several strategies may be considered, including surface PEGylation, polymer coating, and carboxylate-rich surface modifiers.

Indomethacin encapsulated within β-CD cavities may increase hydrophobic domains at the particle surface (20). Electron microscopy images indicated a monolithic, trapezoidal, cubic structure of the synthesized nanocomposites.

The XRD, FTIR, and BET results showed successful dual-drug loading in the nanocomposite without undesirable changes in the structure of the drugs or nanocomposites. These results also indicated the presence of some hydrostatic interactions and weak electrochemical interactions. Reported loading efficiencies for tetracycline in MOFs range between 20% and 45% (37), while indomethacin loading in cyclodextrin systems is often 15% - 35% (12). The loading efficiency in this study falls within these reported ranges, suggesting competitive encapsulation capacity.

The sustained release observed in this study is consistent with dual encapsulation mechanisms, including diffusion-controlled release of tetracycline from MOF pores and host-guest dissociation of indomethacin from β-CD cavities. In similar MOF systems, release kinetics frequently follow Higuchi or Korsmeyer-Peppas models, indicating diffusion-dominated or anomalous transport mechanisms. Although kinetic modeling was not explored in depth in the current work, preliminary data suggest a controlled diffusion profile rather than burst release, which is beneficial for wound management (18).

Future studies should incorporate kinetic modeling to determine whether the system follows Fickian diffusion, non-Fickian transport, or erosion-controlled release.

Additionally, the absence of cytotoxicity and the lack of inhibitory effects on cell proliferation indicate the biocompatibility of the Zn_2_(BDC)_2_(DABCO)-β-CD system as a novel drug delivery system. This nanocarrier demonstrated therapeutic potential in mouse skin wound healing and reduced inflammatory markers while enhancing epithelial regeneration, suggesting applicability to advanced combination-therapy systems. This observation aligns with emerging reports of other MOF-based wound dressings as promising nanoplatforms for multidrug delivery in regenerative medicine.

### 5.1. Conclusions

The Zn_2_(BDC)_2_(DABCO)-β-CD MOF nanocomposite co-loaded with tetracycline and indomethacin and incorporated into a topical ointment was successfully formulated for dual antibacterial and anti-inflammatory wound therapy. Physicochemical characterization confirmed structural integrity after drug loading, sustained release, and suitable particle properties for topical delivery. Biological evaluation showed minimal cytotoxicity, improved wound closure, decreased IFN-γ levels, and increased IL-10 levels in treated mice. These findings support the potential of this dual-drug MOF nanocomposite as a promising topical drug delivery platform for wound healing.

## Data Availability

The dataset presented in the study is available on request from the corresponding author during submission or after publication.
